# Additions, Losses, and Rearrangements on the Evolutionary Route from a Reconstructed Ancestor to the Modern *Saccharomyces cerevisiae* Genome

**DOI:** 10.1371/journal.pgen.1000485

**Published:** 2009-05-15

**Authors:** Jonathan L. Gordon, Kevin P. Byrne, Kenneth H. Wolfe

**Affiliations:** 1 Smurfit Institute of Genetics, Trinity College, Dublin, Ireland; 2 Department of Plant Systems Biology, VIB, Ghent, Belgium; University of Michigan, United States of America

## Abstract

Comparative genomics can be used to infer the history of genomic rearrangements that occurred during the evolution of a species. We used the principle of parsimony, applied to aligned synteny blocks from 11 yeast species, to infer the gene content and gene order that existed in the genome of an extinct ancestral yeast about 100 Mya, immediately before it underwent whole-genome duplication (WGD). The reconstructed ancestral genome contains 4,703 ordered loci on eight chromosomes. The reconstruction is complete except for the subtelomeric regions. We then inferred the series of rearrangement steps that led from this ancestor to the current *Saccharomyces cerevisiae* genome; relative to the ancestral genome we observe 73 inversions, 66 reciprocal translocations, and five translocations involving telomeres. Some fragile chromosomal sites were reused as evolutionary breakpoints multiple times. We identified 124 genes that have been gained by *S. cerevisiae* in the time since the WGD, including one that is derived from a hAT family transposon, and 88 ancestral loci at which *S. cerevisiae* did not retain either of the gene copies that were formed by WGD. Sites of gene gain and evolutionary breakpoints both tend to be associated with tRNA genes and, to a lesser extent, with origins of replication. Many of the gained genes in *S. cerevisiae* have functions associated with ethanol production, growth in hypoxic environments, or the uptake of alternative nutrient sources.

## Author Summary

Genomes evolve in structure as well as in DNA sequence. We used data from 11 different yeast species to investigate the process of structural evolution of the genome on the evolutionary path leading to the bakers' yeast *S. cerevisiae*. We focused on an ancestor that existed about 100 million years ago. We were able to deduce almost the complete set of genes that existed in this ancestor and the order of these genes along its chromosomes. We then identified the complete set of more than 100 structural rearrangements that occurred as this ancestor evolved into *S. cerevisiae* and found that some places in the genome seem to be fragile sites that have been broken repeatedly during evolution. We also identified 124 genes that must be relatively recent additions into the *S. cerevisiae* genome because they were not present in this ancestor. These genes include several that play roles in the unique lifestyle of this species, as regards the intensive production and consumption of alcohol.

## Introduction

Inferring the genome organization and gene content of an extinct species has the potential to provide detailed information about the recent evolution of species descended from it. If we know what was present in the genome of an ancestor, we can deduce how a current-day descendant differs from it. We can then ask questions about how it came to be different. The most recent changes in a genome are often the most interesting ones, because they reflect the most recent (or even current) evolutionary pressures acting on that genome [1],[2].

Yeast species offer the potential for the precise reconstruction of ancestral genomes, because many genomes have been sequenced and they show extensive colinearity of gene order among species [3]–[6]. As the number of sequenced genomes from related species rises, so does the precision with which we can reconstruct their history. In this study we compare the genomes of a group of species in the subphylum Saccharomycotina, spanning an evolutionary time-depth that is comparable to that of the vertebrates [7]. A whole-genome duplication (WGD) event occurred during the evolution of this subphylum [8], and we can compare the genomes of several species (including *S. cerevisiae*) that are descended from this event to the genomes of several species that branched off before the WGD occurred. We focus on an ancestor that existed approximately 100–200 Mya, at the point immediately before the WGD occurred. The evolutionary period beginning with this ancestor corresponds to a time during which the *S. cerevisiae* lineage became increasingly adapted to rapid fermentative growth [9],[10] and extensive rearrangement of the genome occurred (including the deletion of thousands of redundant copies of duplicated genes) [11].

Previous studies in other systems have employed both manual and computational approaches to reconstructing ancestral genomes. One of the most successful applications of computational methods has been the estimation of the ancestral order of orthologous genes in the common ancestor of 12 Drosophila species [12],[13]. Ancestral reconstruction is more difficult when ancient polyploidizations are present [14]. In studies of the 2R duplications in vertebrates, for example, the emphasis has been on establishing the ancestral gene content of paralogous chromosomal regions rather than on their precise gene order [15],[16]. We chose to use a manual, parsimony-based, approach to reconstructing the yeast ancestor at the point of WGD. The manual approach has the attractions of being tractable (whereas computational methods are still under development [17],[18]), of providing an independent result to which computational results can be compared, and of forcing us to examine every rearrangement event without prejudice as to what mechanism might have caused it.

Sankoff and colleagues [14],[17],[18] have developed computational methods that aim to reconstruct ancestral gene order in datasets that include polyploidizations. In recent work [18], they evaluated their ‘guided genome halving’ (GGH) algorithm by comparing its results to ours, using a preliminary version of the manually-derived ancestral yeast gene order that we report here as a ‘gold standard’. As currently implemented, the GGH algorithm can only consider input from a single post-WGD genome and 1–2 non-WGD outgroups, and only considers genes that are duplicated in the post-WGD genome.

Inferring the set of genes that existed in a yeast ancestor, and the order of those genes along the chromosomes, is of interest from both genome-evolutionary and organismal-evolutionary standpoints. Knowing the ancestral gene order enables us to trace all the inter- and intra-chromosomal rearrangements that occurred *en route* from this ancestor to the current *S. cerevisiae* genome, which is informative about the molecular mechanisms of evolutionary genome rearrangement and is also phylogenetically informative. Knowing the ancestral gene content allows us to identify genes that have been added to, or lost from, the *S. cerevisiae* genome during the past 100 Myr. Previous studies have shown that changes in gene content can provide a strong indication of changing evolutionary circumstances, either in cases of gene loss (such as the losses of *GAL*, *DAL* and *BNA* genes in *Candida glabrata* [1],[19],[20]) or in cases of gene gain (such as the *ADH2* and *URA1* genes of *S. cerevisiae* [9],[21],[22]). Even though it may not be possible to conclude that any particular gene gain was adaptive, the clear links between the functions of the gained genes *ADH2* and *URA1* and the adaptation of *S. cerevisiae* to a fermentative lifestyle [23] suggested to us that a systematic search for all the genes that were gained by *S. cerevisiae* since WGD would be worthwhile.

## Results/Discussion

### Ancestral Genome Reconstruction

**Figure 1:**
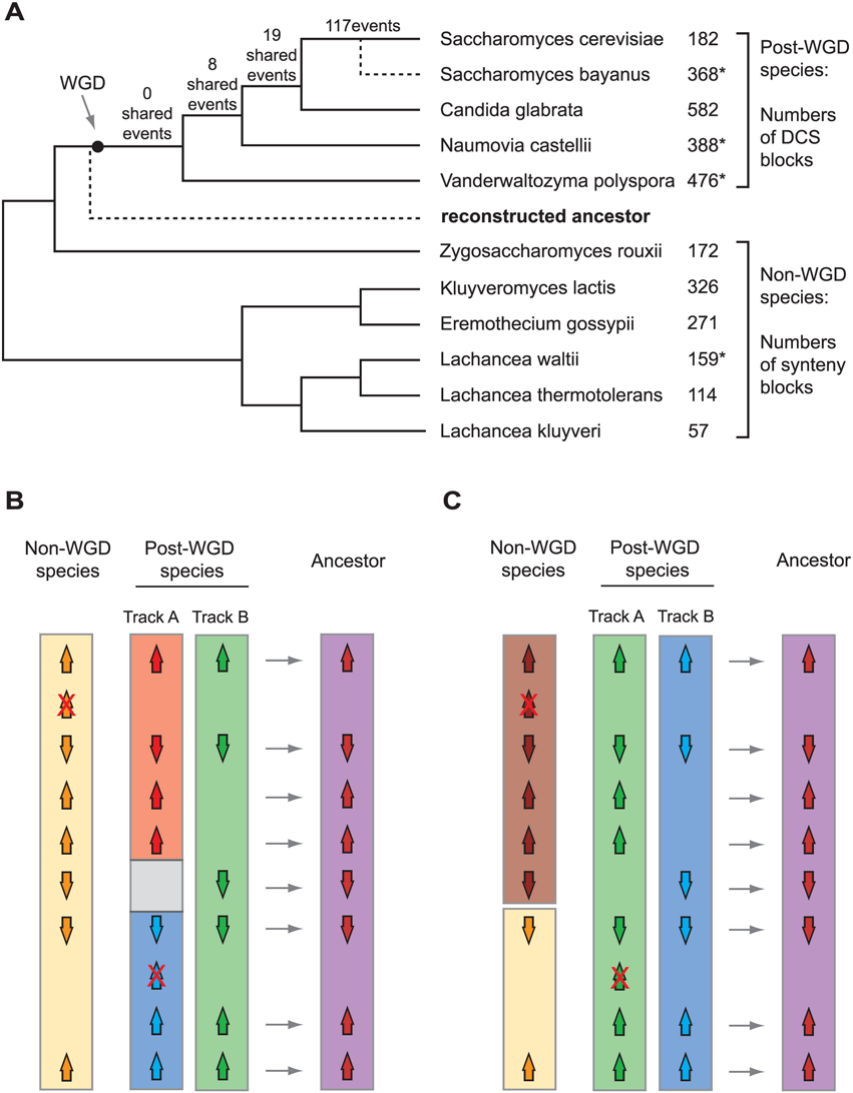
Inferring ancestral gene content and gene order. (A) Phylogenetic relationships among the species considered (not to scale; topology from ref. [89]), and the position of the ancestor whose genome was reconstructed. The dot indicates the whole-genome duplication. Numbers of genomic rearrangements (reciprocal translocations and inversions) shared by post-WGD clades are shown above the branches (Table S2). The numbers on the right show the number of genomic blocks shared by each species and the reconstructed ancestor. Asterisks indicate block numbers that are probably overestimates because the corresponding genome sequences are fragmented into many contigs. (B,C) Principles of the parsimony method for ancestral genome reconstruction. Colors represent continuous chromosomal regions. For simplicity the diagrams show only one non-WGD species and one post-WGD species, but in practice we used all the species shown in panel A. We infer that a gene was present in the ancestor if it is present in at least one non-WGD species and one track of a post-WGD species, or if paralogs are present on both post-WGD tracks. Genes found only on one post-WGD track, or only in non-WGD species, cannot be inferred to have been present in the ancestor and are marked with red crosses. Two types of scenario for gene order rearrangement can exist [26]. In each case the inferred ancestral order is shown on the right. (B) Single break of synteny. Gene order is conserved between a non-WGD species and one of the two tracks in a post-WGD species, due to a rearrangement on the other track after WGD. (C) Double break of synteny. The two tracks from the post-WGD species agree with each other but disagree with the non-WGD species. The ancestor is inferred to have the gene order present in the post-WGD species.

We used a manual parsimony approach to reconstruct the gene order and gene content of the yeast ancestor that existed immediately prior to WGD (Figure 1). The reconstruction was made by visually comparing the local gene orders in every region of the genome, stepping through the genome in overlapping 25-gene windows using the Yeast Gene Order Browser [YGOB; 6]. Initially, during 2007–08, we compared data from five post-WGD species (*S. cerevisiae*, *S. bayanus*, *C. glabrata*, *Naumovia castellii* and *Vanderwaltozyma polyspora*) and three non-WGD species (*E. gossypii*, *Kluyveromyces lactis* and *Lachancea waltii*) and inferred an ancestral genome based on these data. Later, in 2009, we added the genomes of three more non-WGD species (*Zygosaccharomyces rouxii*, *L. thermotolerans* and *L. kluyveri* [24]) and re-examined the whole genome window-by-window using YGOB. This process confirmed that our initial ancestral reconstruction was largely correct, but identified a few places where the gene content or local gene order in the ancestor needed to be revised. In particular, by adding data from more non-WGD species we were able in some cases to detect non-WGD orthologs of *S. cerevisiae* genes that are short and rapidly-evolving, which previously appeared to be unique to *S. cerevisiae* (for example, *YLR146W-A*).

The gene order and content of the ancestor were inferred as shown in Figure 1B,C. We first established the gene content, and then examined the adjacency relationships among these genes. Within any post-WGD species such as *S. cerevisiae*, most of the genome can be sorted into pairs of sister regions that have a double-conserved synteny (DCS) relationship with any non-WGD species such as *L. waltii* [25],[26]. Breaks in the DCS pattern correspond to two types of event, called single-breaks and double-breaks of synteny [26]. For each single-break of synteny (Figure 1B), because we have genome sequences from multiple post-WGD species, and because the endpoints of the chromosomal rearrangements in different species generally do not coincide, we can infer the species and chromosomal track on which the break happened. This inference also tells us the ancestral gene order across the site of breakage: in general, for a single break, the ancestral order has been disrupted in one track in one post-WGD species, but it is still conserved in the same track from the other post-WGD species, in the sister track from all the post-WGD species, and in the non-WGD species. Similarly for each double-break of synteny (Figure 1C), because we have multiple genome sequences from non-WGD species we can in general identify the break as having occurred in one particular non-WGD species. A small number of double-breaks of synteny are caused by situations where all the non-WGD species show one gene order but both of the tracks from all the post-WGD species show a different order. These breaks correspond to rearrangements that occurred on the branch between the *Z. rouxii* divergence and the common ancestor of the post-WGD species (before the WGD happened). We do not include these breaks in our analysis because we are only interested in events that occurred after the WGD.

**Figure 2:**
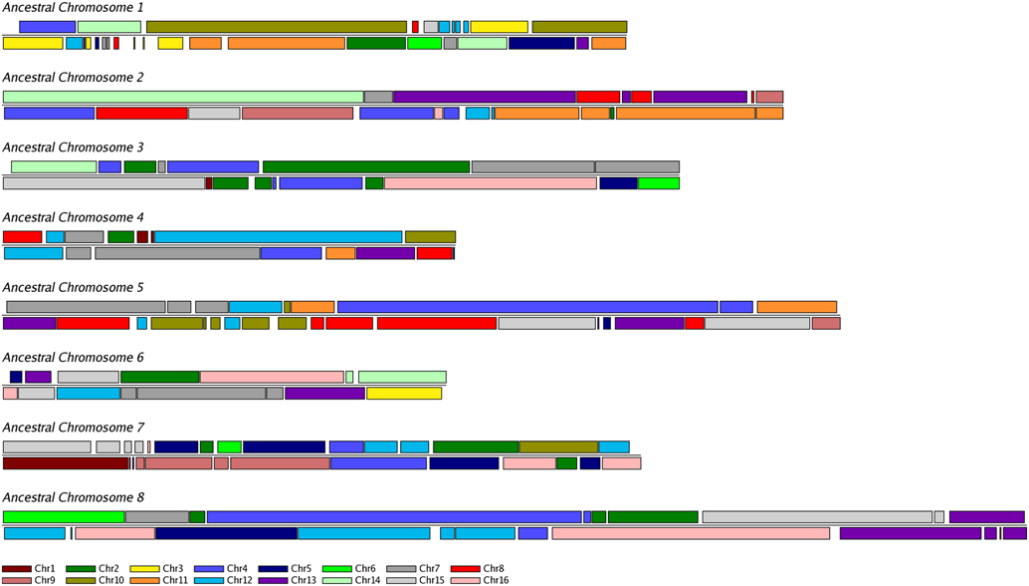
Synteny relationship between the reconstructed ancestral genome and the modern *S. cerevisiae* genome. Each colored block represents a region in *S. cerevisiae* that is colinear with a region of the ancestral genome. The 182 double-conserved-synteny blocks in *S. cerevisiae* map onto the ancestor in a 2∶1 pattern. Colors correspond to the 16 modern *S. cerevisiae* chromosomes as shown at the bottom.

Manual reconstruction by this method resulted in an inferred ancestral genome with eight chromosomes, containing 4703 protein-coding genes. The ancestral gene set represents the intersections of orthologous genes between non-WGD and post-WGD species, and between ohnologs (paralogs formed by WGD) across the post-WGD species. The ancestral genome is listed in Table S1 and can be browsed using YGOB (http://wolfe.gen.tcd.ie/ygob). Genes in this genome were given names such as Anc_1.125, meaning the 125^th^ gene on chromosome 1 of the ancestor. The ancestral gene set accounts for 5158 (92%) of the 5601 genes currently present in *S. cerevisiae* (1088 ohnologs and 4070 single copy genes), which covers all genomic regions in *S. cerevisiae* except for the subtelomeric regions (discussed below). The *S. cerevisiae* genome can be mapped onto the inferred ancestral genome in 182 DCS blocks that tile together in an unambiguous 2∶1 fashion across the ancestral genome (Figure 2). Similarly, the other post-WGD species and non-WGD species can be mapped onto the ancestral genome, with 2∶1 and 1∶1 mappings, respectively, by the numbers of blocks shown in Figure 1A. The *C. glabrata* genome is much more rearranged (582 blocks) than *S. cerevisiae* as previously noted [19],[27]. The *L. kluyveri* genome is remarkably unrearranged, with the whole genome mapping into just 57 blocks relative to the ancestor.

Our inferred ancestral genome is incomplete in some regards:

- The reconstructed gene order and content covers only the internal regions of chromosomes, extending out to the approximate borders of the subtelomeric regions where synteny conservation stops. The gene content in subtelomeric regions is different in every species and the dynamic nature of these regions makes it impossible to trace evolutionary events over long timescales [28],[29]. In *S. cerevisiae*, for example, a total of 299 genes are located in subtelomeric regions that lie beyond the ends of the ancestral reconstruction. These genes make up the majority of the 443 *S. cerevisiae* genes that do not have counterparts in the ancestral genome (the others are 124 gained genes that will be discussed later, and 20 transpositions as described in the next paragraph).
- We only included a gene in the ancestral genome if we could be confident about its location at the time of WGD, so genes that transposed at approximately the same time as the WGD are not included. An example is *DAL1*, which is single-copy in all genomes but at a different site in the post-WGD and non-WGD species [30]. Twenty *S. cerevisiae* genes fell into this category; we did not count them as gains because they were probably present in the ancestral genome, but we do not know where.
- We cannot detect genes that may have been present in the ancestor at the moment of WGD but were subsequently lost, in both copies, by all the post-WGD species considered. The *MAT*a2 HMG domain gene is a possible example [31],[32].
- It can be difficult to determine whether fast-evolving genes are orthologous. There are a few points in the genome where we can identify a group of rapidly-evolving orthologs among post-WGD species, and a group of rapidly-evolving orthologs among non-WGD species, but we cannot establish whether the two groups are themselves orthologous. An example is *S. cerevisiae YJL144W* (with orthologs in four post-WGD species) and *L. thermotolerans KLTH0F05478g* (with orthologs in four non-WGD species), both of which lie in the interval between Anc_1.205 and Anc_1.206, and have similar sizes and transcriptional orientation but no significant sequence similarity.
- The local gene order in the ancestor is uncertain in a few places, because none of the extant species retains all of the relevant genes.

### Rearrangement Route to the Current *S. cerevisiae* Genome

**Figure 3:**
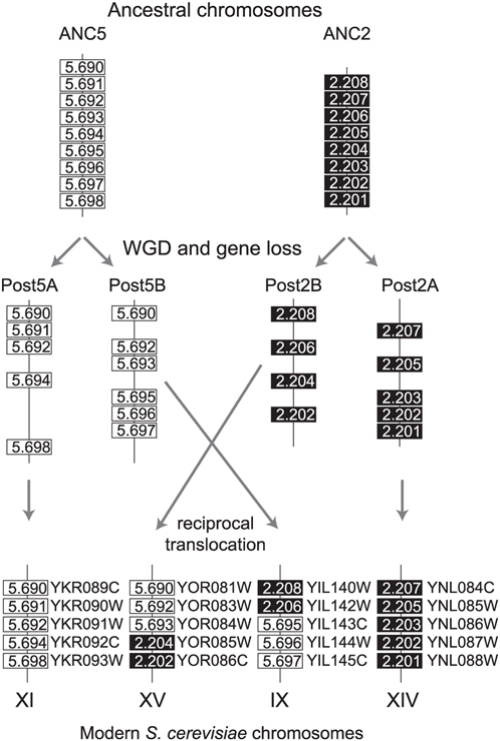
Example of a simple reciprocal translocation in *S. cerevisiae*. Parts of two ancestral chromosomes, ANC5 and ANC2, are shown at the top. After WGD, these formed four chromosomes (labeled Post5A, Post5B, Post2A, Post2B), each of which retains a subset of the ancestral gene sets. Parts of *S. cerevisiae* chromosomes XI and XIV are derived from chromosomes Post5A and Post2A, respectively, without further rearrangement. A reciprocal translocation between chromosomes Post5B and Post2B gave rise to part of *S. cerevisiae* chromosomes XV and IX.

Using the breakpoints between *S. cerevisiae* synteny blocks in the ancestral genome, we inferred the large scale chromosomal rearrangements that have occurred in the *S. cerevisiae* lineage since the WGD. Most rearrangement events could be classified as either reciprocal translocations (Figure 3) or inversions. Note that it is impossible to count inversions and reciprocal translocations with absolute precision, because if a genomic region that contains one endpoint of a reciprocal translocation subsequently undergoes inversion, the result is identical to one that could be produced by two successive reciprocal translocations (Figure S1). We counted these situations as two reciprocal translocations, so we have probably misclassified some inversions as reciprocal translocations. Inversions were defined as events where the two endpoints of the rearrangement were on the same ancestral chromosome and on the same post-WGD track.

**Figure 4:**
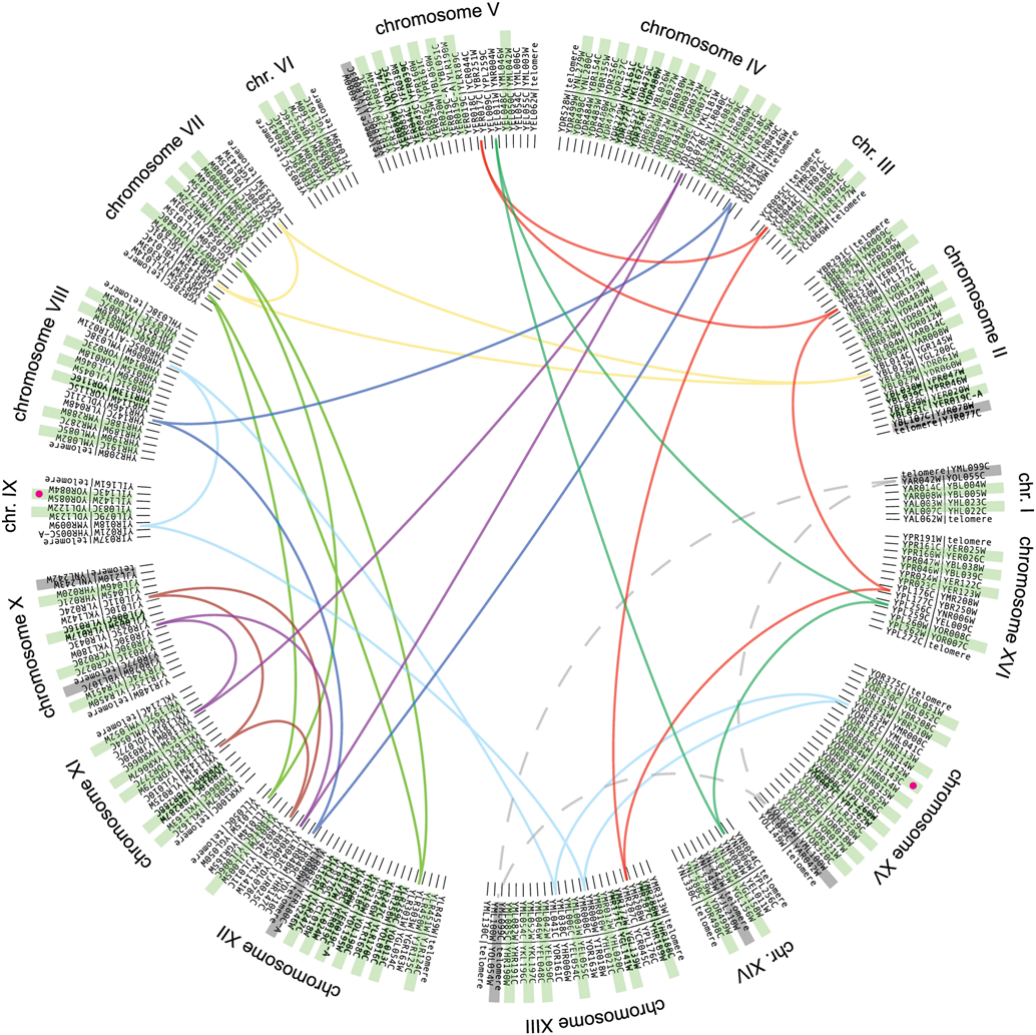
Reciprocal translocations that formed the *S. cerevisiae* genome from the ancestral genome. Each point on the circle represents a breakpoint, and names two genes (separated by a | symbol) that were adjacent in the ancestral genome but became separated by reciprocal translocation on the *S. cerevisiae* lineage. These breakpoints form the ends of the synteny blocks shown in Figure 2. The genes at the breakpoints are arranged according to their current positions on the *S. cerevisiae* chromosomes, so each breakpoint appears twice in the circle (once for each end, usually on different chromosomes). Green backgrounds join the names of pairs of breakpoints that were formed by simple reciprocal translocation events. As an example, the pink dots highlight the new junctions on chromosomes IX and XV that were formed by the simple reciprocal translocation shown in Figure 3, involving the breakpoints *YOR084W*|*YIL143C* and *YOR085W*|*YIL142W*. In cases of breakpoint reuse, the genes on one side of the pair of breakpoints are adjacent in *S. cerevisiae*, but the genes on the other side are not. By iteratively linking each of the non-matching genes to the gene that is adjacent to it in the *S. cerevisiae* genome, we can describe groups of 3–5 reciprocal translocation events with breakpoints that have been used more than once (colored arcs). We observed one event where a breakpoint created by a telomeric translocation was reused (dashed gray line). Telomeric translocation events are indicated by gray backgrounds on breakpoint names. This diagram is an adjacency graph [90] applied to a genome halving context [91],[92].

In total we inferred 73 inversion events and 66 reciprocal translocations events on the evolutionary path from the ancestor to *S. cerevisiae* (Table 1). Five of the inversions have endpoints at telomeres. There were also five non-reciprocal translocations, which we call ‘telomeric translocations’ because they involved an exchange between a telomere and an internal region of another chromosome, which moved the end of an arm from one chromosome to another (one of these events occurs at a shared inversion/translocation breakpoint). The data indicate that some intergenic regions were re-used as breakpoints in more than one rearrangement event. We classified the rearrangements as consisting of 34 simple inversion events (not overlapping other inversions or reusing breakpoints), 39 complex inversion events (overlapping other rearrangements and/or reusing breakpoints), 44 simple reciprocal translocation events, and 22 reciprocal translocation events involving breakpoint reuse (Figure 4). These results are in reasonable agreement with our estimate from a decade ago of 70–100 rearrangement events, based only on *S. cerevisiae* data [33].

**Table 1:** Breakpoint re-use in *S. cerevisiae*.

Event type	Number of events	Number of breakpoints		Re-use ratio^a^
		Expected	Observed	
Reciprocal translocations	66	132	118	1.12
Inversions	73	141^b^	126	1.16
Telomeric translocations	5	5	5	1.00
All events	144	278	228^c^	1.22

a Ratio of expected to observed breakpoints.

b Five inversion events occur at telomeres, so each makes only one breakpoint.

c 21 breakpoints are shared by a reciprocal translocation and an inversion.

If some post-WGD species share a rearrangement relative to the ancestor but others retain the ancestral gene order, the rearrangement event is a phylogenetically informative character [34],[35]. We searched for rearrangements shared by any pair of post-WGD species. As described below, we found many that support the branching order of the post-WGD species shown in Figure 1A (Table S2). We did not find any shared rearrangements supporting alternative topologies. This result supports our previous conclusion, based on shared patterns of gene losses, that *N. castellii* is an outgroup to a clade containing *C. glabrata* and *S. cerevisiae* [11],[36]. In contrast, phylogenies based on sequence analysis tend to place *C. glabrata* outside *N. castellii* and *S. cerevisiae* [1],[37],[38], a result that we believe is an artifact.

Given this phylogeny, the post-WGD species define four temporal intervals for rearrangements (Figure 1A): (*i*) no rearrangements are shared by all the post-WGD species relative to the ancestor; (*ii*) 8 rearrangement events are shared by *N. castellii*, *C. glabrata* and *S. cerevisiae* (6 inversions, 1 reciprocal translocation, 1 telomeric translocation); (*iii*) 19 rearrangements are shared only by *C. glabrata* and *S. cerevisiae*, with *N. castellii* and *V. polyspora* retaining the ancestral organization (13 inversions, 6 reciprocal translocations); and (*iv*) 117 rearrangements are unique to *S. cerevisiae* or shared by this species and *S. bayanus* (54 inversions, 59 reciprocal translocations, 4 telomeric translocations). Most of the rearrangements that are specific to *S. cerevisiae* are temporally ambiguous relative to each other. We did not subdivide the group of 117 events into those that occurred before and after the *S. bayanus* divergence because the *S. bayanus* genome assembly is quite fragmented. The above analysis does not include gene transpositions, which we find to be relatively rare in yeast genomes but which are difficult to count precisely because to identify a transposed gene in a particular species, we need to be certain that it is orthologous to a gene at a non-syntenic location in the ancestral genome.

The lack of rearrangements in the first time interval is notable because it indicates that *V. polyspora* separated from the other lineages soon after the WGD. We also found that no rearrangements occurred on one genomic track of all the post-WGD species, relative to the other track and the ancestor, prior to this speciation. This observation argues against the possibility that the WGD event was an allopolyploidization rather than an autopolyploidization (see ref. [11] for discussion): if it was an allopolyploidization, then the two hybridizing genomes must have been completely colinear.

### Breakpoint Re-Use and Properties of Breakpoint Sites

It was necessary to infer breakpoint reuse at the ends of some synteny blocks. Reused breakpoints appear as cycles in the map of breakpoint pairs (Figure 4). The evolutionary re-use of breakpoints has previously been identified in studies on mammals and *Drosophila* [13],[39]. We find that for both reciprocal translocations and inversions, there are fewer breakpoints than expected if every event had unique ends (Table 1). The average number of breaks per used site is 1.12 for reciprocal translocations and 1.16 for inversions. Some sites were used as endpoints of both an inversion and a reciprocal translocation, and if we pool these two categories there are only 228 unique breakpoints instead of the expected 278, implying an average of 1.22 breaks per site (Table 1).

**Figure 5:**
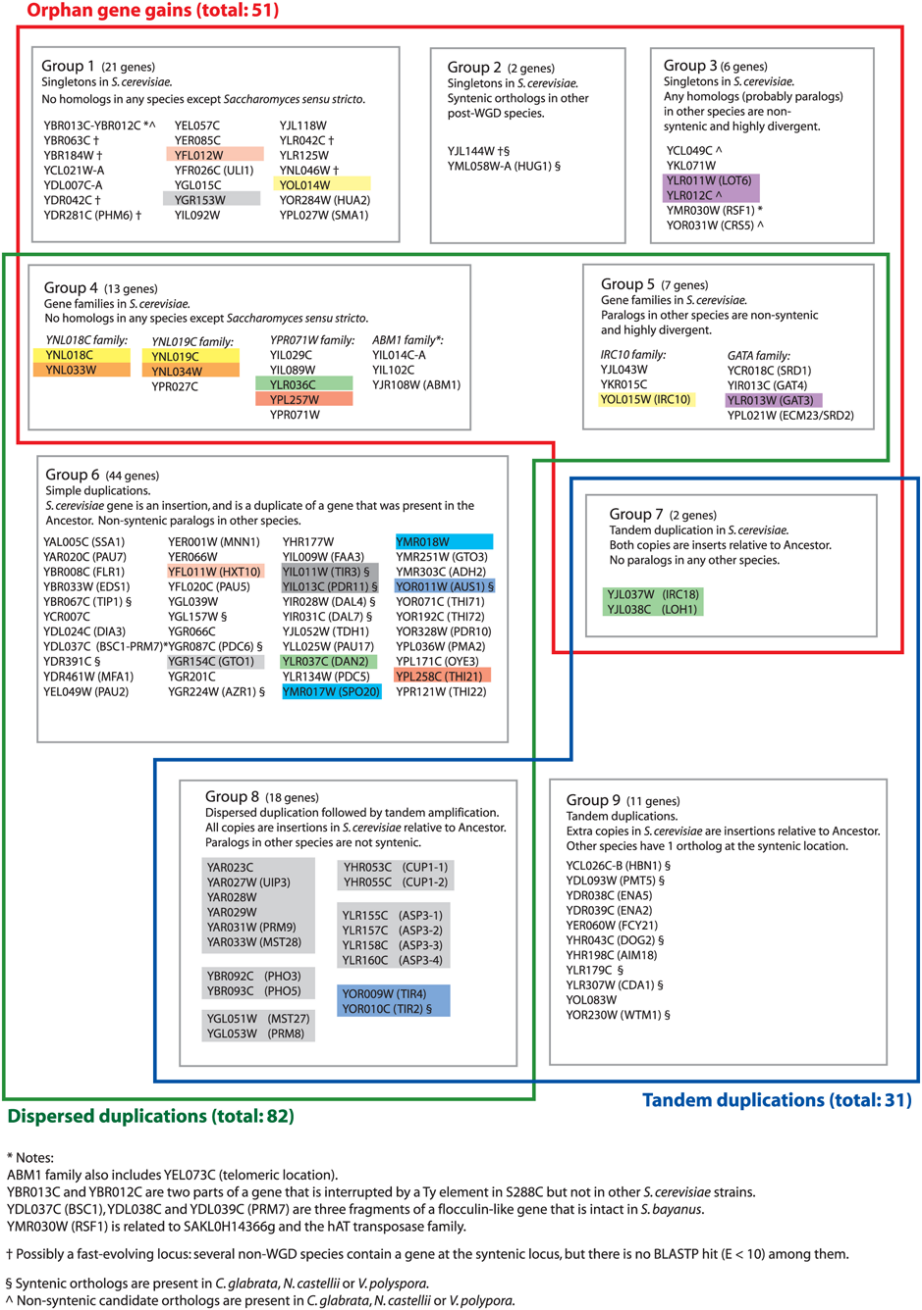
The 124 non-telomeric genes that were gained on the *S. cerevisiae* lineage since WGD. Colored backgrounds indicate genes that are adjacent and may have been gained simultaneously.

We identified 96 sites in the ancestral genome at which genes are inferred to have been gained subsequently in the lineage leading to *S. cerevisiae*. The total number of gained genes is 124, because some sites contain groups of consecutive gained genes (Figure 5). We were surprised to find that 33 (34%) of these ‘gene gain’ sites are beside tRNA genes. tRNA genes have previously been linked to sites of genomic rearrangement between *E. gossypii* and *S. cerevisiae* [26]. Furthermore, it is known that origins of replication in yeast are often located near tRNA genes [40], and it seems plausible that origins might be fragile sites for evolutionary breakage and/or integration of new DNA. We used computer simulation to test the significance of the associations among tRNA genes, origins of replication, evolutionary breakpoints, and sites of gene gain (see *Methods*). tRNA genes are present at breakpoints and gain sites about three times more often than expected by chance (Table 2, rows 2 and 3), and origins are present about twice as often (Table 2, rows 4 and 5). It should be noted however that the locations of all the tRNA genes are known whereas it is probable that many origins have not yet been identified [41].

**Table 2:** Significance of observed colocalized elements in *S. cerevisiae*.

Row	Pattern of colocalized elements				Observed number in genome	Simulation mean	Proportion of simulations≥Observed	*P*_FDR_
	tRNA	Origin	Breakpoint	Gene gain				
1	**+**	**+**	−	−	31	11.5	2×10^−6^	4.4×10^−6^
2	**+**	−	**+**	−	32	9.7	1×10^−6^	1.1×10^−5^
3	**+**	−	−	**+**	13	4.0	0.00018	0.00032
4	−	**+**	**+**	−	19	10.6	0.0096	0.015
5	−	**+**	−	**+**	9	4.4	0.032	0.035
6	−	**−**	**+**	**+**	6	3.8	0.17	0.19
7	**+**	**+**	**+**	−	15	0.5	1×10^−6^	5.5×10^−6^
8	**+**	**+**	−	**+**	9	0.2	1×10^−6^	3.7×10^−6^
9	**+**	−	**+**	**+**	11	0.2	1×10^−6^	2.8×10^−6^
10	−	**+**	**+**	**+**	2	0.2	0.018	0.022
11	**+**	**+**	**+**	**+**	1	0.0	0.0098	0.013

*P*_FDR_, statistical significance after correction for false discovery rate.

There are several plausible mechanisms by which tRNA genes could precipitate genomic rearrangements. tRNA genes exist in multiple near-identical copies in the genome, so illegitimate recombination between these sequences could result in reciprocal translocations [42],[43]. Ty retroelements tend to integrate beside tRNA genes and provide long sections of near-identical sequence scattered around the genome that could be substrates for ectopic recombination, as seen in *S. cerevisiae* irradiation experiments [44]. Ty LTRs, tRNA genes, and origins of replications have also all been associated with the endpoints of spontaneous segmental DNA duplications in *S. cerevisiae* [45]. Replication forks tend to stall near highly-expressed genes (such as tRNA genes), and sites of replication fork collapse are hotspots for chromosomal rearrangements [46],[47]. It is also possible that the Ty-encoded reverse transcriptase has played a direct role in the integration of new genes into sites beside tRNA genes, similar to the way that cDNA fragments of transcribed genes are sometimes captured at sites of double-strand break repair in *S. cerevisiae* experiments [48].

### Categories of Gained Genes

We identified 124 genes, excluding those in subtelomeric regions, that are inferred to have been gained on the lineage leading to *S. cerevisiae* during the time since WGD (Figure 5). The *S. cerevisiae* gene set that we used in this study consists only of genes that are conserved between *S. cerevisiae* and at least one of the other *Saccharomyces sensu stricto* species (*dN*/*dS* ratio<1 in the analysis of Kellis *et al.* [49]), or that are duplicates of other genes in *S. cerevisiae* (again with *dN*/*dS*<1), so we can be confident that the all the gains we identity are real genes and not annotation artifacts. Some of the gained genes are unique to *S. cerevisiae* and *sensu stricto* species, while others are shared by the other post-WGD species (Figure 5).

The 124 gained genes range from those with high similarity to another gene in the *S. cerevisiae* genome to those with no similarity to any known gene from any organism. We classified the gained genes into nine groups as described in Figure 5, and then into three larger categories according to their apparent mechanism of formation. The three large categories are:

- *Dispersed duplications*, where a progenitor gene that remains in a conserved syntenic context became duplicated to produce a new gene at a new site in the genome, and so appears as an insertion relative to the ancestral order. An example is *ADH2*, which was made by duplication of *ADH1*. For the simple dispersed duplications in group 6 of Figure 5, the availability of the ancestral genome reconstruction allows us to identify which gene of the pair is the parent of the other.
- *Tandem duplications*, such as the array of three Na^+^ ion transporters *ENA1*, *ENA2* and *ENA5* (group 9). In this example the ancestral genome has one *ENA* gene at the syntenic location, so we arbitrarily designated *ENA2* and *ENA5* as ‘new’ genes in *S. cerevisiae*. For some other *S. cerevisiae* tandem arrays (such as the *CUP1* array and others in group 8) there is no gene at the syntenic location in the ancestral genome. Many of these arrays are known to be polymorphic in size among strains of *S. cerevisiae* [50],[51].
- *Orphan gene gains*, where a gene that appears as an insertion relative to the ancestral gene order has no apparent progenitor. Some orphans (groups 4, 5 and 7) are members of orphan families, where all members of the family are insertions relative to the ancestor. In some cases (groups 3 and 5) orphan genes contain an identifiable protein domain but there is no other gene that is similar enough in sequence to be regarded as a possible progenitor of the orphan. It is possible that some of the loci we have classified as orphan gene gains are in fact older genes that were present in the ancestral genome but are evolving so rapidly that the homology between the post-WGD and the non-WGD sequences is unrecognizable; some loci where this situation may apply are marked in Figure 5.

### Functions of Genes Gained in the *S. cerevisiae* Lineage Since WGD

Analysis of the functions of the gained genes should provide insight into the evolutionary pressures that have acted on *S. cerevisiae* in the period since WGD but, remarkably, there is no functional information in the *Saccharomyces* Genome Database (SGD) for almost half of the recently gained genes. None of the 124 genes is essential when deleted, according to SGD. The non-essentiality of gained genes is not surprising because they were gained by an organism that was already fully functional in its environment before they were gained. It is particularly notable that only 16 of the 51 orphans in Figure 5 have been assigned genetic names, which would indicate that something is known about their function.

In the sections below, we discuss some of the functional groups of gained genes. The gene information in these sections is derived primarily from summaries in the SGD and YPD databases [52],[53], and from a MIPS (Munich Information Centre for Protein Sequences) catalog analysis.

### Lifestyle Adaptations

#### Ethanol production and consumption

Thomson *et al.* [9] identified a group of recently-duplicated genes in *S. cerevisiae* that are implicated in that species' ecological strategy of making, accumulating and consuming ethanol. This strategy enables *S. cerevisiae* to out-compete other microorganisms by monopolizing the available resources of glucose. *S. cerevisiae* is able to take up glucose rapidly because it can achieve a high flux through glycolysis, which occurs because pyruvate (the end product of glycolysis) is not allowed to accumulate [54],[55]. Pyruvate is converted to ethanol and excreted from the cell, instead of entering the relatively slow step of import into the mitochondria and respiration via the TCA cycle. Later, when external glucose is exhausted, the ethanol can be re-imported and respired. This make-and-consume strategy provides a competitive advantage, but at a cost of a slightly lower net yield of ATP per glucose molecule. There is also a risk that ethanol excreted by a cell might not be recovered, due to evaporation or consumption by other cells. Thomson *et al.* [9] identified the duplication that formed *ADH2* as the key step in the development of this strategy, and identified five other gene family expansions that they suggested were also involved and occurred at about the same time. Our approach is independent of Thomson *et al.*'s (using synteny rather than a molecular clock), and our results confirm that several of the genes they identified – *ADH2*, *PDC5*, *TDH1*, *PHO3*, *PHO5* – were all added to the *S. cerevisiae* genome in the time since the WGD. We also identify a second pyruvate decarboxylase gene, *PDC6*, as a recent gain, and the *PHO3/PHO5*-related gene *DIA3*.

#### Thiamin uptake and biosynthesis

Thiamin diphosphate (ThDP) is an essential cofactor of decarboxylase enzymes, including the pyruvate decarboxylases (the ancestral gene *PDC1* and the gained genes *PDC5* and *PDC6*) that function in ethanol production. Our gained gene set includes at least six genes that are involved in the ThDP pathway. In this pathway, the precursor thiamin phosphate (TP) is converted to the intermediate molecule thiamin (vitamin B1), which in turn is converted to the biologically active molecule ThDP [56]. Two gained genes, *THI21* and *THI22*, code for enzymes that synthesize TP. Two more, *PHO3* and *PHO5* (and possibly also *DIA3*), code for extracellular acid phosphatases that dephosphorylate TP found outside the cell to form thiamin, which can then be imported. Two other gained genes, *THI71* and *THI72*, have been characterized as transporters of thiamin. Interestingly, *S. cerevisiae* and the *sensu stricto* species are prototrophic for thiamin, whereas the other post-WGD species considered here are not [57],[58], which is likely a result of these multiple recent gene gains. *THI71* ( = *NRT1*) also functions as a high-affinity transporter of nicotinamide riboside, a precursor in NAD biosynthesis [59]. As ThDP is essential in many metabolic pathways, the gain of additional transporters of exogenous thiamin and of biosynthesis genes may represent an important shift in the physiology of *S. cerevisiae*. One possibility, suggested by Thomson *et al.* [9] in the context of the *PDC* and *PHO* gene duplications, is that selection for increased ethanol production resulted in increased demand for thiamin as a cofactor of pyruvate decarboxylase.

#### Hypoxic growth

Several previous studies have suggested that *S. cerevisiae* has become increasingly adapted towards growth in conditions of low oxygen [23],[30],[60], and the set of gained genes includes several (in addition to the *ADH*, *PDC* and *THI* duplications) whose presence can be interpreted in this context. One way in which *S. cerevisiae* has adapted is by reducing its dependence on biochemical pathways that use molecular oxygen. The genes *DAL4* and *DAL7* were gained (by duplication) during a reorganization of the purine degradation pathway to eliminate an oxygen-requiring step [30]. Oxygen is also required for the biosynthesis of sterols, which are an essential component of membranes [61]. Two of the gained genes, *AUS1* and *PDR11*, are ABC transporters that play major roles in the uptake of sterols from outside the cell under anaerobic conditions [62].

### Gene Family Expansions

#### Escaped members of subtelomeric gene families

Most yeasts have species-specific gene family amplifications in their subtelomeric regions [28],[29],[63]. In *S. cerevisiae* the major subtelomeric families are the *DAN/TIR*, *PAU* and *DUP240* families. We were unable to reconstruct the gene order in the subtelomeric regions of the ancestral genome, so our reconstruction excludes most members of these families. Nevertheless, the set of recent gains in *S. cerevisiae* includes some members that became relocated to internal sites on chromosomes. There are nine members of the *DUP240* family in the gained set, including six in a tandem array on chromosome I. The *DUP240* genes are nonessential membrane spanning proteins that have been suggested to function in membrane trafficking [64]. The gained genes *PAU2*, *PAU5*, *PAU7*, *PAU17*, *DAN2*, *TIP1*, *TIR2* and *TIR4* all code for either cell wall or integral membrane proteins, and are induced by anaerobiosis [65],[66]. *TIP1*, *TIR2*, *TIR4* and *DAN2* all appear to function in the remodeling of the cell wall and plasma membrane in response to altered environmental conditions such as anaerobiosis or cold shock [66],[67].

#### Rapidly evolving families of unknown function

**Figure 6:**
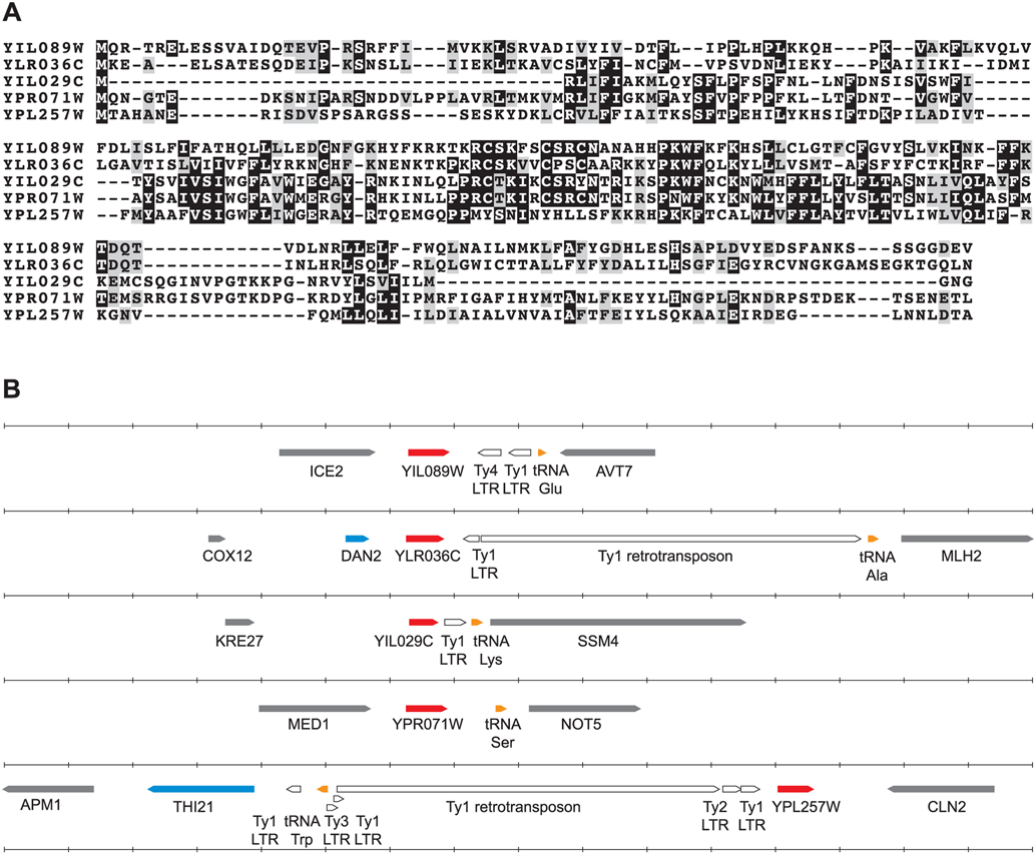
The *YPR071W* gene family in *S. cerevisiae*. All members of this family were gained by *S. cerevisiae* since WGD and all are located near tRNA genes. (A) T-Coffee multiple alignment of the five proteins. Black and grey backgrounds show residues that are identical or similar, respectively, in ≥3 sequences. (B) Maps of the genomic regions around each gene. Red, *YPR071W* family members; orange, tRNA genes; blue, other genes in the gained set; white, Ty elements and long terminal repeats; gray, genes in the ancestral set (only the first gene on each side is shown). Tick marks indicate intervals of 1 kb.

The gained gene set includes some uncharacterized and highly divergent families of orphan genes (groups 4 and 5 in Figure 5). The largest is a family of five genes related to *YPR071W* (Figure 6). There is no obvious progenitor for this family in the ancestral genome, and there are no homologs in the NCBI databases outside the *sensu stricto* species. None of the five has a phenotype when deleted [52]. The family is highly divergent, with only 10% amino acid sequence identity between the most divergent pair (Figure 6A); they can be recognized as a family because *YPR071W* hits the four other members in a BLASTP search (E≤0.004). Interestingly, all five genes are located beside tRNA genes (Figure 6B). We also identified two other orphan families with similarly high levels of divergence and no apparent progenitor. Each of these families contains one member that has been given a genetic name (*ABM1*, with an aberrant microtubules phenotype, and *IRC10* with a possible DNA recombination phenotype; [68],[69]). The mechanism by which these families originated and diversified is unclear.

#### GATA family transcription factors

Another rapidly evolving family of gained genes contains transcription factors with GATA zinc finger domains [70]. Four of the nine proteins with this domain in *S. cerevisiae* have been gained since WGD (Figure 5). *ECM23* is involved in cell wall morphogenesis and may be a negative regulator of genes for pseudohyphal growth [71]. *SRD1* was identified as a suppressor of a mutation in *RRP1*, which codes for a nucleolar protein involved in rRNA maturation [72]. Very little is known about the functions of *GAT3* and *GAT4* [73]. Curiously, the functions of the other (non-gained) GATA factors in *S. cerevisiae* are much better defined; they are four regulators of nitrogen metabolism (*GLN3*, *GAT1*, *DAL80*, *DEH1*) [74] and the *ASH1* repressor of *HO* endonuclease [75].

### Other Changes

#### *RSF1*, a domesticated transposable element

One of the gained genes, *RSF1* (*YMR030W*), is required for respiratory growth on glycerol (a non-fermentable carbon source) [76]. Rsf1 plays a role in transcriptional changes that occur during the metabolic shift from (fermentative) glycerol synthesis to (respiratory) glycerol catabolism [77], and may be a transcription factor [78] or a protein that interacts with transcription factors [77]. We find that *RSF1* is a truncated member of a gene family that exists in about five copies in *V. polyspora* (e.g., *Kpol_387.6*) and *N. castellii* (e.g., *Scas_707.15d*), and in two copies in *L. kluyveri* (*SAKL0H14366g* and *SAKL0H03916g*). PSI-BLAST searches show that these proteins in the other yeast species are related to the hAT family of DNA transposons [79]. The hAT family is ubiquitous in plants, animals, and filamentous fungi but has not been previously reported to be present in Saccharomycotina species [80]. The *V. polyspora* and *N. castellii* members of the family are integrated at species-specific sites and show higher sequence similarity within species than between species, consistent with them being transposable. *RSF1* is integrated at a site that is specific to the *Saccharomyces sensu stricto* species. It codes for a protein of only 376 amino acids, corresponding to the N-terminus of a protein that is typically ∼900 amino acids in the other yeasts, and does not retain the region with highest similarity to the hAT family. The part retained in *RSF1* is likely to be the DNA-binding domain, though we could not find the BED finger DNA-binding motif that is present at the N-terminus of some hAT proteins [81],[82].

Importantly, *RSF1* is conserved at the same location in *S. cerevisiae* and *S. bayanus* and has a *dN*/*dS* ratio of 0.22, which indicates that its protein sequence is subject to selective constraint even in the absence of transposition. We suggest that *RSF1* is derived from a hAT element, but was recruited to perform a cellular function in *S. cerevisiae*. Sequence polymorphism in *RSF1* has been found to be one of the main causes of variation in sporulation efficiency among natural isolates of *S. cerevisiae*, which could place the gene under strong selection although some null alleles have also been identified [78]. The *L. kluyveri* gene *SAKL0H03916g* may be a second (older) example of a domesticated hAT transposon [81], because orthologs of this gene have been retained in a syntenic location in many yeast species (including the *S. cerevisiae* WGD pair *VID22* and *YGR071C*).

#### Other gained genes

Also notable among the gained genes are several involved in catabolism of alternative nitrogen sources (asparaginases *ASP3-1*, *ASP3-2*, *ASP3-3*, *ASP3-4*; cytosine transporter *FCY21*; *DAL4* and *DAL7* in the allantoin pathway), ion homeostasis (*ENA2* and *ENA5* for Na^+^ efflux, and *PMA2* for H^+^ efflux), drug resistance (*AZR1* and *FLR1* in the MFS superfamily, and *AUS1*, *PDR10* and *PDR11* in the ABC superfamily), defense against oxidative stress (glutathione S-transferases *GTO1* and *GTO3* [83], the *LOT6* sensor of redox state [84], and *OYE3* which detoxifies small α,β unsaturated aldehydes such as acrolein, a product of oxidative attack on lipids [85]), and two genes (*YGL039W*, *YGL157W*) coding for oxidoreductases related to *GRE2*.

#### Genes lost in *S. cerevisiae*

At 88 loci in the ancestral genome, *S. cerevisiae* retained neither of the gene copies after WGD. We know that they were present in the ancestor because they have been retained syntenically in at least one other post-WGD species and in at least one non-WGD species. Most of these losses involve genes of unknown function, but among the others are some that can be related to changes in the physiology of *S. cerevisiae*, such as loss of the oxygen-requiring enzyme D-amino acid oxidase [30] and the loss of *URA9* which was displaced by *URA1*, so decoupling uracil biosynthesis from mitochondrial respiration [21]. Other *S. cerevisiae*-specific losses are more puzzling, such as its loss of an ortholog of the *K. lactis* high-affinity glucose/galactose transporter *HGT1* (*KLLA0A11110g*) [86].

### Conclusion

Reconstructing the content and gene order of the ancestral yeast genome just prior to WGD has provided a mechanism for studying the structural rearrangements that occurred subsequent to WGD. Our reconstruction is dependant on the set of extant genomes available for comparison, so it is likely that our list of candidate gene gains includes some false positives that will turn out to have been present at the time of WGD. As more genome sequences become available the ancestral gene set will become progressively more complete and the list of gains may shrink.

From a biological perspective, the main shortcoming of our work is that we were unable to reconstruct the telomeric regions of the genome, corresponding to the last ∼10 genes on each arm of each chromosome in *S. cerevisiae*. These regions turn over so dynamically that synteny breaks down almost completely between the species considered here. This is unfortunate because many of the most interesting evolutionary events such as the gain of genes by horizontal gene transfer (HGT) from other species [22], seem to occur preferentially near telomeres. Our set of candidate gene gains in *S. cerevisiae* contains only two possible cases of gene gain by HGT at internal chromosomal sites (*YLR011W/LOT6* and *YLR012W*; we did not study these in detail), whereas Hall *et al.* [22] found eight examples of apparent transfer of bacterial genes into telomeric sites. A second shortcoming is that we relied on sequence conservation (*dN*/*dS*<1) among the *sensu stricto* species as a way of distinguishing between genuine *S. cerevisiae* genes and annotation artifacts, which had the inadvertent effect that we overlooked any genes that may have been gained by *S. cerevisiae* in the time since it diverged from the other *sensu stricto* species; one such case is *BSC4*, which appears to have been formed *de novo* in *S. cerevisiae* [87].

The set of genes inferred to have been gained on the *S. cerevisiae* lineage is relatively small (2% of the gene set) and their functions point squarely towards increasing adaptation to the ‘fermentative lifestyle’ [23]. They indicate increasing throughput of the glycolysis and fermentation pathways, and adaptation towards growth in conditions with little oxygen, including modifications to the cell wall and the bypass of biochemical pathways that require molecular oxygen by importing substances from outside the cell. There are also many gained genes in our set that we have not discussed in detail here because they did not fall into larger functional groups. Further analysis of these gains on an individual basis may reveal insights into the evolution of *S. cerevisiae* and the other species in the WGD clade.

## Methods

### Nomenclature

In this paper we have adopted the revised genus nomenclature proposed by Kurtzman [88]: *Saccharomyces castellii* becomes *Naumovia castellii*; *Kluyveromyces polysporus* becomes *Vanderwaltozyma polyspora*; *Ashbya gossypii* becomes *Eremothecium gossypii*; *Kluyveromyces waltii* becomes *Lachancea waltii*; *Kluyveromyces thermotolerans* becomes *Lachancea thermotolerans*; and *Saccharomyces kluyveri* becomes *Lachancea kluyveri*. In this scheme each genus name refers to a monophyletic group, whereas previously *Saccharomyces* and *Kluyveromyces* were polyphyletic. We did not change any gene names, even though in many species the gene names have a prefix that is an acronym of the obsolete species name.

### Counting DCS and Synteny Blocks

The numbers of double-conserved synteny (DCS) and synteny blocks between the reconstructed ancestor and each other species (Figure 1A) were counted automatically using an algorithm that smoothes over small inversions and other interruptions in cases where endpoints are ≤20 genes apart in the ancestral genome. For *S. cerevisiae* our manual analysis identified 228 breakpoints (Table 1 and Figure 4), which subdivide the 16 linear chromosomes into 244 segments. The discrepancy in numbers between these 244 segments and the 182 DCS blocks in *S. cerevisiae* (Figures 1A and 2) is due to the use of the smoothing algorithm.

### Evolution of Ancestral Organization into *S. cerevisiae* Gene Order

We described the inferred ancestral gene order in terms of synteny blocks of current *Saccharomyces cerevisiae* genes. We manually identified intrachromosomal rearrangements (inversions) between the ancestor and *S. cerevisiae* and reversed them, revising our synteny blocks, in order to more easily identify the endpoints of reciprocal translocations. For each synteny block end not at a telomere, the location is at a position in the ancestral genome that underwent a reciprocal translocation in its transition towards the current *S. cerevisiae* genome. Each synteny block end in the ancestral genome is bordered by another synteny block found elsewhere in the current *S. cerevisiae* genome. The two breakpoints at the ends of two ancestral synteny blocks now adjacent in the current *S. cerevisiae* genome were created by a reciprocal translocation event that joined them together from different ancestral locations. Concurrently the other synteny blocks that border each breakpoint in the ancestral genome were joined together by the same event. We ordered the synteny blocks in the manner in which they are found along each chromosome in *S. cerevisiae*, thus inferring all the interchromosomal rearrangements between the ancestral polyploid genome and *S. cerevisiae* (Figure 4). To confirm that the inferred reciprocal translocation events were correct, we found the location in *S. cerevisiae* of the other synteny block ends joined by each event. In cases where these synteny blocks are not adjacent in the *S. cerevisiae* genome, we found the ancestral breakpoint locations of the blocks that are adjacent to each of these blocks in *S. cerevisiae*, inferring another reciprocal translocation event. If the synteny blocks bordering each breakpoint were again not adjacent, this process was repeated.

### Inference and Analysis of Probable Gene Gains in the *S. cerevisiae* Lineage since the WGD

To obtain a set of likely gene gains (Figure 5) we subtracted the set of *S. cerevisiae* genes represented in the ancestral genome from the curated set of 5601 *S. cerevisiae* genes currently used in YGOB. YGOB's *S. cerevisiae* gene set is based on the SGD annotation (‘verified’ and ‘uncharacterized’ protein-coding loci only) with some additional manual curation. It omits loci that failed Kellis *et al.*'s test of reading frame conservation among *sensu stricto* species [49]. *S. cerevisiae* genes that are present in YGOB set but absent from the inferred ancestral set are candidates for having been gained in the *S. cerevisiae* lineage after the WGD. We did not include subtelomeric genes from the YGOB set, as orthologous relationships across species break down at the telomeres [49]. This candidate set of gains was then manually checked to ensure that there were no possible non-syntenic homologs that were ancestral but missing from our ancestral genome reconstruction due to a breakdown of synteny information. Any cases where a good candidate non-syntenic homolog was found were removed from the gained set and flagged as a likely transposition event. It is possible that the set of candidate gained genes may also contain ancestral genes that were lost in all of the non-WGD species used here but have orthologs in more distantly related outgroups.

### Testing for Coincidence of tRNAs, Origins, Breakpoints, and Gene Gains

We compiled lists of the 245 *S. cerevisiae* intergenic regions that contain one or more tRNA genes [from SGD; 52], the 228 intergenic regions that contain evolutionary breakpoints on the *S. cerevisiae* lineage, the 96 sites of gene gain in *S. cerevisiae*, and 267 intergenic regions that contain an origin of replication in *S. cerevisiae* (from OriDB [41]; we included origins that overlap with genes). We counted the numbers of intergenic regions that contain combinations of multiple types of site.

We then used computer simulation to estimate the significance of the observed numbers of coinciding sites (Table 2). In each of 1 million replicates we simulated a genome with 5100 intergenic spacers (the estimated number of intergenic spacers between *S. cerevisiae* genes that are at an ancestral locus). We placed the same numbers of tRNA genes, origins, breakpoints, and gene gain sites as above into randomly chosen spacers in the simulated genome. Each type of site was placed randomly and independently of the other types of site. We then counted the numbers of spacers containing all possible combinations of types of site in the replicate. Finally, we compared the observed numbers of coinciding sites in the real data to the distribution of results from the simulation (Table 2). The proportion of simulated genomes in which the number of sites with a particular colocalization pattern matches or exceeds the observed number of such sites in the real genome is an empirical measure of the statistical significance of the observation, under the null hypothesis of a random distribution of sites. We then applied a false discovery rate correction to these empirical P-values.

## Supporting Information

Figure S1 Some inversions can be indistinguishable from reciprocal translocations. Numbers 1–8 represent genomic segments, and minus symbols indicate inverted orientation. The upper part shows the effect of a reciprocal translocation (RT) followed by an inversion (inv) of a region that includes one endpoint of the RT. The lower part shows a scenario of two consecutive RTs. The two scenarios produce the same final order of genomic segments, so it is not possible to tell which scenario is correct.
(0.24 MB PDF)

Table S1 Excel file listing the genes of the reconstructed ancestral genome in order through its 8 chromosomes, and their orthologs in modern genomes.
(1.87 MB XLS)

Table S2 Genomic rearrangements shared among post-WGD species.
(0.02 MB XLS)
