# Supplementary material for: Additions, Losses, and Rearrangements on the Evolutionary Route from a Reconstructed Ancestor to the Modern Saccharomyces cerevisiae Genome
Source: PLoS Genet. 2009 May 15;5(5):e1000485. doi: 10.1371/journal.pgen.1000485 (PMC2675101; doi:10.1371/journal.pgen.1000485)
Supplement: Figure S1 — Some inversions can be indistinguishable from reciprocal translocations. Numbers 1–8 represent genomic segments, and minus symbols indicate inverted orientation. The upper part shows the effect of a reciprocal translocation (RT) followed by an inversion (inv) of a region that includes one endpoint of the RT. The lower part shows a scenario of two consecutive RTs. The two scenarios produce the same final order of genomic segments, so it is not possible to tell which scenario is correct. (0.24 MB PDF) [file pgen.1000485.s001.pdf]

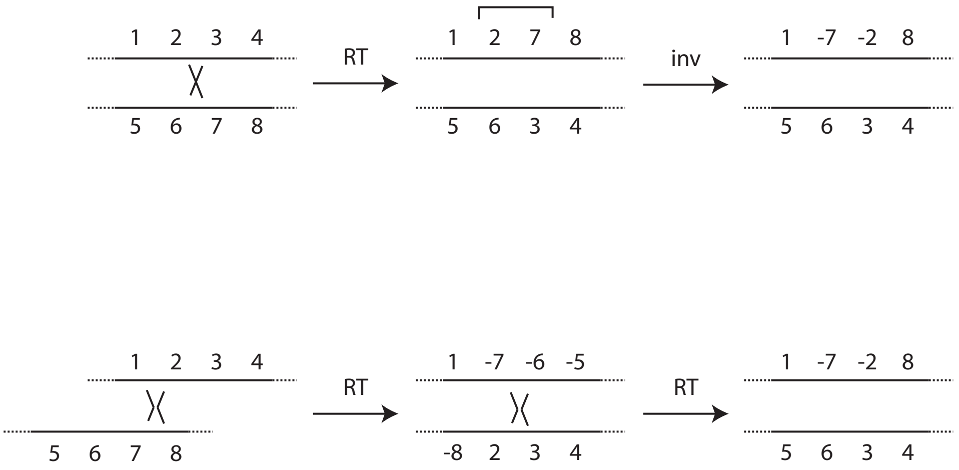

Figure S1. Some inversions can be indistinguishable from reciprocal translocations. Numbers 1-8 represent genomic segments, and minus symbols indicate inverted orientation. The upper part shows the effect of a reciprocal translocation (RT) followed by an inversion (inv) of a region that includes one endpoint of the RT. The lower part shows a scenario of two consecutive RTs. The two scenarios produce the same final order of genomic segments, so it is not possible to tell which scenario is correct.
